# Fyn nanoclustering requires switching to an open conformation and is enhanced by FTLD-Tau biomolecular condensates

**DOI:** 10.1038/s41380-022-01825-y

**Published:** 2022-10-18

**Authors:** Ramón Martínez-Mármol, Christopher Small, Anmin Jiang, Tishila Palliyaguru, Tristan P. Wallis, Rachel S. Gormal, Jean-Baptiste Sibarita, Jürgen Götz, Frédéric A. Meunier

**Affiliations:** 1 Clem Jones Centre for Ageing Dementia Research (CJCADR), Queensland Brain Institute (QBI), The University of Queensland, St Lucia Campus, Brisbane, QLD 4072 Australia; 2 University of Bordeaux, CNRS, Interdisciplinary Institute for Neuroscience, IINS, UMR 5297, F-33000 Bordeaux, France; 3 School of Biomedical Sciences, The University of Queensland, St Lucia Campus, Brisbane, QLD 4072 Australia

**Keywords:** Neuroscience, Cell biology

## Abstract

Fyn is a Src kinase that controls critical signalling cascades and has been implicated in learning and memory. Postsynaptic enrichment of Fyn underpins synaptotoxicity in dementias such as Alzheimer’s disease and frontotemporal lobar degeneration with Tau pathology (FTLD-Tau). The FLTD P301L mutant Tau is associated with a higher propensity to undergo liquid–liquid phase separation (LLPS) and form biomolecular condensates. Expression of P301L mutant Tau promotes aberrant trapping of Fyn in nanoclusters within hippocampal dendrites by an unknown mechanism. Here, we used single-particle tracking photoactivated localisation microscopy to demonstrate that the opening of Fyn into its primed conformation promotes its nanoclustering in dendrites leading to increased Fyn/ERK/S6 downstream signalling. Preventing the auto-inhibitory closed conformation of Fyn through phospho-inhibition or through perturbation of its SH3 domain increased Fyn’s nanoscale trapping, whereas inhibition of the catalytic domain had no impact. By combining pharmacological and genetic approaches, we demonstrate that P301L Tau enhanced both Fyn nanoclustering and Fyn/ERK/S6 signalling via its ability to form biomolecular condensates. Together, our findings demonstrate that Fyn alternates between a closed and an open conformation, the latter being enzymatically active and clustered. Furthermore, pathogenic immobilisation of Fyn relies on the ability of P301L Tau to form biomolecular condensates, thus highlighting the critical importance of LLPS in controlling nanoclustering and downstream intracellular signalling events.

## Introduction

**Fig. 1:**
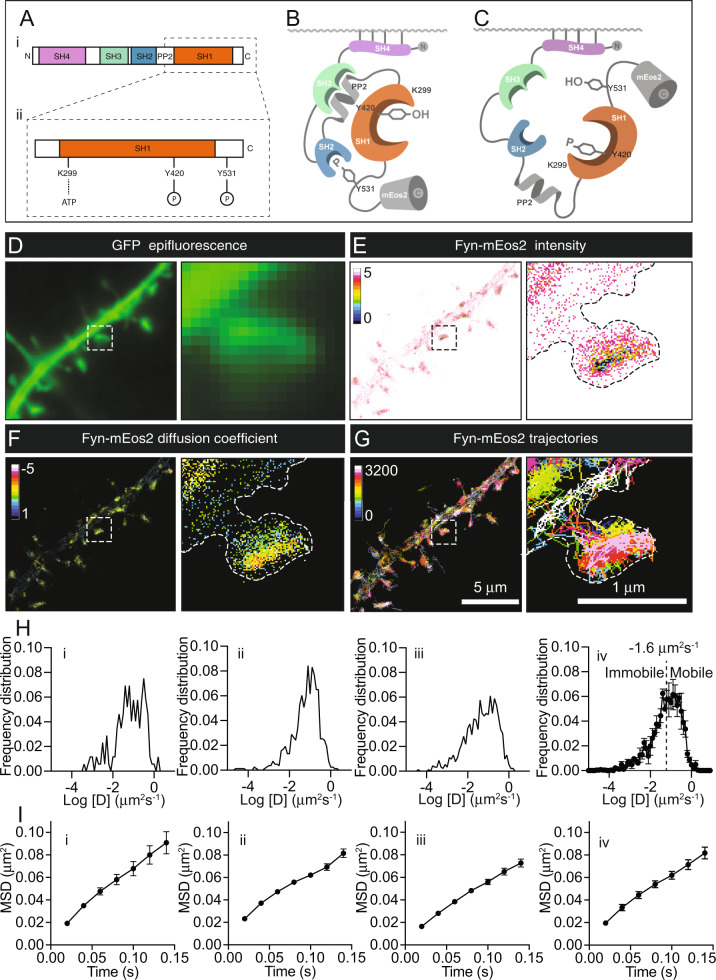
Single-molecule tracking photoactivated localisation microscopy (sptPALM) of Fyn-mEos2. **A** Illustration showing (i) the protein domains of Fyn (SH1-4 and PPII), with the boxed outline magnified below in (ii) to highlight key epitopes in the SH1 domain (K299 and Y420) and C-terminal tail (Y531) of Fyn. **B** Tertiary structure of Fyn in its closed, inactive conformation. mEos2 is conjugated to the C-terminus of Fyn. **C** Tertiary structure of Fyn in its open, active conformation. mEos2 is conjugated to the C-terminus of Fyn. **D**–**G** SptPALM of Fyn-mEos2 co-transfected with GFP in secondary dendritic branches and spines of hippocampal neurons (DIV19-22). Panels depict representative **D** GFP epifluorescence image, **E** localisation intensity map, **F** diffusion coefficient map, and **G** trajectory map for Fyn-mEos2. Note that cooler colours within the intensity and the diffusion coefficient maps in (**E**) and (**F**) designate regions of higher localisation intensities and mobility, respectively. Boxed outlines in the left panels are shown magnified on the right. **H**(**i–iii**) Examples of frequency distribution of Fyn-mEos2 diffusion coefficients [D] (plotted as Log_10_ [D] (µm^2^s^−1^)) from individual neurons. **H**(**iv**) Average Fyn-mEos2 frequency distribution of diffusion coefficients from (**i**), (**ii**) and (**iii**). The threshold separating immobile and mobile molecules (dotted line) was set at −1.6 µm^2^s^−1^ [49]. **I**(**i**–**iii**) Examples of average mean-square displacement (MSD; µm^2^) curves over time (0.14 s) of trajectories from individual neurons. **I**(**iv**) Average Fyn-mEos2 MSD from (**i**), (**ii**) and (**iii**). Error bars are standard errors of the mean (SEM).

Fyn is a member of the Src family of kinases (SFKs), a group of enzymes that regulate signal transduction by catalysing the phosphorylation of tyrosine residues. Fyn is expressed in numerous cell types, including lymphocytes, neurons and glia. Like other SFKs, Fyn is an intracellular, membrane-associated enzyme that has four conserved motifs known as Src homology domains (SH1, SH2, SH3 and SH4) (Fig. 1A). The C-terminal SH1 domain is the catalytic domain responsible for the phosphorylation of tyrosine residues of target proteins and is connected to the SH2 domain via a polyproline type II (PPII) helix linker (Fig. 1A–C). The SH2 and SH3 domains in the middle of the protein control the interaction of Fyn with its substrates. Finally, the N-terminal SH4 domain is responsible for the association of Fyn with the plasma membrane, which is mediated through its myristylation and palmitoylation, thereby facilitating the interaction between Fyn and its membrane-associated substrates [1].

The kinase activity of Fyn is controlled by its transition between an inactive-closed (contracted) conformation that is unable to bind substrates (Fig. 1B), and an active-open (extended) conformation that can bind to its substrates and execute its catalytic activity (Fig. 1C). The inactive-closed conformation of Fyn is stabilised by two intramolecular interactions. The first intramolecular interaction occurs between the SH2 domain and the phosphorylated Y531 epitope (in the human sequence of Fyn) within Fyn’s C-terminal domain, locking Fyn in an inactive folded state that prevents the interaction of the kinase with its associated substrates [2, 3]. The second interaction occurs between the hydrophobic surface of the SH3 domain and the proline and hydrophobic rich motifs (P-X-X-P motifs) of the PPII helix linker [2], further locking the enzyme in its inactive folded conformation. Conversely, dephosphorylation of the Y531 residue converts Fyn into its open conformation, resulting in its priming. This intermediate extended, primed form of Fyn is then activated by trans-autophosphorylation of the Y420 residue, located at the central ‘activation loop’ present within the catalytic SH1 domain [4]. In this open conformation, the SH2 and SH3 domains are accessible to the medium (Fig. 1C), allowing for their interaction with external ligands [5].

Fyn is responsible for integrating multiple signalling cascades. In neurons, Fyn facilitates cell-to-cell communication by promoting the scaffolding of the N-methyl-D-aspartate (NMDA) receptor through phosphorylation of the NR2B subunit of the receptor at the Y1472 epitope. This phosphorylation increases the affinity of NR2B for postsynaptic density protein 95 (PSD95) and thereby stabilises NMDA receptor clusters at the membrane enabling them to maintain postsynaptic excitatory currents [6]. Fyn is also implicated in neurodegeneration, as overactive Fyn is believed to exacerbate cell death by promoting excess activation of NMDA receptors, leading to aberrant calcium entry into the postsynapse and neuronal excitotoxicity [7]. Furthermore, Fyn has been shown to play a critical role in mediating neurotoxic signalling in Alzheimer’s disease (AD) and frontotemporal lobar degeneration with Tau pathology (FTLD-Tau) [8, 9]. Fyn promotes neurotoxicity downstream of both the amyloid-β (Aβ) peptide, which forms extracellular amyloid plaques, and the microtubule-associated protein Tau, which in a hyperphosphorylated form accumulates into neurofibrillary tangles (NFTs), a key hallmark of AD and FTLD-Tau [10]. In transgenic mouse lines that replicate AD pathology, the knockout (KO) of Fyn protects against the loss of presynaptic terminals and delays premature mortality [11]. Conversely, overexpression of constitutively active Fyn leads to hyperactivity and premature death [7]. Hippocampal slices from Fyn KO mice have been shown to resist the toxic effects of treatment with toxic Aβ oligomers [12]. Similarly, pharmacological inhibition of Fyn rescues the behavioural deficits observed in AD mice [13], whereas its overexpression exacerbates the neuronal deficits present in AD mice [14]. Fyn also plays a central role in Tau-mediated pathology [9]. Fyn interacts through its SH2 domain with Tau phosphorylated at the Y18 epitope [15], an early marker for the formation of NFTs in AD patients [16, 17]. Fyn also interacts through its SH3 domain with P-X-X-P motifs located in the proline-rich region of Tau [18, 19]. Subcellular compartmentalisation of Fyn and Tau appears to be crucial for their toxicity. Mis-localisation of Tau into dendritic spines mediates the synaptic dysfunction associated with AD and FTLD-Tau [20, 21], and Tau itself has a role in targeting Fyn to dendrites, where Fyn mediates Aβ toxicity [22].

Increasing evidence suggests that key molecules, including Fyn, are organised into nanodomains or nanoclusters, which are nanometre-sized regions that serve as functional platforms for different molecules to concentrate and integrate signalling cascades [23, 24]. Changes in the organisation of receptors and signalling molecules within such nanodomains are emerging as a key regulator of neuronal toxicity [23]. Several proteins involved in AD display an aberrant clustering associated with the pathology [24]. The metabotropic glutamate receptor 5, for example, exhibits increased clustering when engaged by Aβ [25], and the amyloid precursor protein (APP) from which Aβ is derived organises into regulatory nanodomains that control the availability of APP molecules for proteolytic processing [26]. In this respect, Tau has been shown to control the lateral trapping of Fyn into nanoclusters in hippocampal dendrites and their spines, with single-molecule imaging of Fyn-mEos2 revealing enhanced mobility and decreased clustering in Tau KO neurons [27]. Tau functions as a neuronal scaffold protein with multiple roles including the progression of neurodegenerative diseases [8]. Expression of an FTLD mutant Tau (P301L) aberrantly increases the number of Fyn nanoclusters in spines, via an unknown mechanism which likely contributes to NMDA-mediated excitotoxicity [27]. Recently, liquid–liquid phase separation (LLPS) has been suggested as a novel mechanism that controls the organisation of neuronal proteins into clusters [28, 29]. LLPS has been described as the reversible compartmentalisation of intracellular components through membraneless structures [30]. Typically, multidomain proteins undergo LLPS when they associate with biomolecular condensates by establishing multivalent, low-affinity interactions [31] between intrinsically disordered regions of their own protein domains [32], or those of other binding partners [33]. Both wild-type (WT) Tau and several of its pathological forms, including P301L mutant Tau, organise into LLPS biomolecular condensates in vitro [34–36]. However, several questions remained unanswered: (i) how Tau condensates contribute to the nanoscale organisation of binding partners such as Fyn; (ii) what is the spatiotemporal organisation of Fyn that is involved in its efficient transactivation; and (iii) what are the mechanisms that underly the Fyn-Tau toxic partnership?

In this study, we used single-particle tracking photoactivated localisation microscopy (sptPALM) to determine how the nanoscale organisation of Fyn is affected by its activity and conformation, and by its association with pathologically interacting partners in dendrites of live hippocampal neurons. By combining sptPALM with pharmacological inhibition of Fyn, the use Fyn mutants and a monobody directed against Fyn SH3 domain, we could demonstrate that altering the conformation of Fyn can modulate its mobility dynamics in neurons. Furthermore, we show that the presence of Fyn-interacting proteins such as the FTLD P301L mutant Tau has direct consequences on Fyn immobilisation. Interestingly, perturbation of this direct interaction could not completely restore the mobility of Fyn, suggesting the presence of alternative indirect mechanisms involved in the nanoscale entrapment of Fyn. The combination of imaging approaches with pharmacological interventions and the use of mutants revealed the formation of droplet-like Tau-P301L biomolecular condensates in cells. Specifically, a truncated form of Tau lacking the microtubule-binding repeats (MTBR) region was unable to undergo LLPS and immobilise Fyn, suggesting that the formation of pathogenic Tau biomolecular condensates may be required for the observed further entrapment of Fyn. Taken together, our findings suggest a novel molecular mechanism through which Fyn and Tau interact in the dendritic compartment of neurons to exacerbate the toxic effects of Fyn that are observed in conditions with increased pathological Tau such as FTLD-Tau.

## Materials and methods

### Animal ethics and mouse strains

All experimental procedures were conducted under the guidelines of the Australian Code of Practice for the Care and Use of Animals for Scientific purposes and were approved by The University of Queensland Animal Ethics Committee (QBI/254/16/NHMRC and QBI/254/20/NHMRC). WT mice (C57BL/6J strain) used throughout the study were maintained on a 12-h light/dark cycle and housed in a PC2 facility with ad libitum access to food and water.

### Primary hippocampal cultures

Primary hippocampal neurons were prepared as described previously [27, 37]. Briefly, pregnant dams (C57BL/6J mice) were euthanized using cervical dislocation, from which embryos (E16) were extracted and dissected in 1× Hank’s buffered salt solution, 10 mM HEPES pH 7.3, 100 U/ml penicillin-100 μg/ml streptomycin (GIBCO-Thermo Fisher Scientific). The dissected hippocampal tissue was then digested with trypsin (0.25% for 10 min). Digestion was halted by the addition of foetal bovine serum (FBS) (5%) (GIBCO-Thermo Fisher Scientific) with DNase I (MilliporeSigma) to prevent tissue clumping, and the digested hippocampal tissue was incubated at 37 °C (10 min). Following this, the hippocampal tissue was triturated and centrifuged (120 g, 7 min) and resuspended in neurobasal media, 100 U/ml penicillin-100 μg/ml streptomycin, 1× GlutaMAX supplement (GIBCO-Thermo Fisher Scientific), 1× B27 (GIBCO-Thermo Fisher Scientific) and FBS. Neurons were seeded in poly-L-lysine (MilliporeSigma) coated 29 mm glass-bottom dishes (Cellvis) at a density of 0.8–1 × 10^5^ neurons. A full medium change was performed 2 h after seeding using culturing media (Neurobasal Medium, 100 U/ml penicillin-100 μg/ml streptomycin, 1× GlutaMAX supplement, 1× B27).

### Heterologous cell cultures

HEK-293T cells (ATCC; 293T/17, ATCC®CRL-11268) were maintained in DMEM medium (GIBCO-Thermo Fisher Scientific) supplemented with 10% FBS (GIBCO-Thermo Fisher Scientific), 1× GlutaMAX (GIBCO-Thermo Fisher Scientific) and 100 U/ml penicillin-100 μg/ml streptomycin (MilliporeSigma).

### Plasmids and reagents

The plasmid encoding the FN3 monobody G9 targeted against Fyn’s SH3 domain was a generous gift from Emeritus Professor Brian Kay (University of Illinois at Chicago, Illinois, US). The G9 monobody was subcloned into the pmEos2-N1 plasmid [38] (MB-anti-SH3-mEos2) by inserting BamHI and EcoRI restriction sites at the N- and C-termini of the monobody, followed by BamHI/EcoRI digestion and ligation into the mEos2 vector. Fyn mutant constructs were generated by site-directed mutagenesis, using the QuikChange II site-directed mutagenesis kit (Agilent), and the mEos2-N1 donor vector containing full-length human Fyn isoform 1 [27] as a template. Tau-P301L mutant constructs were generated by site-directed mutagenesis, using the QuikChange II site-directed mutagenesis kit (Agilent), and the pEGFP-N1 donor vector containing the 2N4R human FTLD P301L mutant Tau as a template [27]. All generated plasmids were sequenced by the GRS sequencing facility (Genetic Research Services, The University of Queensland). 3-(4-Chlorophenyl)-1-(1,1-dimethylethyl)-1H-pyrazolo[3,4-d]pyrimidin-4-amine (PP2) and 1-phenyl-1H-pyrazolo[3,4-d]pyrimidin-4-amine (PP3) were purchased from Calbiochem (Merck/Millipore). The aliphatic alcohol 1,6-hexanediol (1,6-HD) was purchased from Sigma (MilliporeSigma).

### Western blotting

HEK-293T cells were seeded in a 6-well plate and co-transfected with lipofectamine 3000 (Thermo Fisher Scientific) according to the manufacturer’s instructions. Transfected cells were incubated for 48 h prior to processing. The following constructs were used: pmEos2-N1 (Addgene #54662), Fyn-mEos2 [27] (human full-length Fyn, isoform 1, fused with mEos2 at the C-teminus), Fyn-Y531F-mEos2 (Fyn-mEos2 containing the Y531F mutation), Fyn-Y531F-K299M-mEos2 (Fyn-mEos2 containing the mutations Y531F and K299M), pCMV-myc, pcDNA6-V5, Fyn-myc [39] (human full-length Fyn, isoform 1, fused with the myc tag at the C-teminus), Tau-P301L-V5 [39, 40] (human tau 2N4R, htau40, containing the FTLD mutation P301L, fused with V5 tag at the C-terminus). Transfected HEK-293T cells were washed twice in ice-cold PBS and lysed in ice-cold lysis buffer (50 mM Tris-HCl, pH 7.4, 150 mM NaCl, 2 mM EDTA, 1% NP-40) supplemented with a protease/phosphatase inhibitor cocktail (Cell Signalling Technology). The lysate was briefly sonicated and centrifuged at 1000 g  for 5 min at 4 °C. The supernatant was then collected, and protein homogenates were boiled at 95 °C in Laemmli buffer. Equal amounts of proteins were loaded and run on a tris-glycine precast gel (4–15%, Bio-Rad) at 150 V and subsequently transferred to a PVDF-FL membrane (Millipore), which was incubated in blocking buffer (Odyssey, TBS). Subsequently, membranes were incubated with primary antibodies. Membranes were stained for total Fyn (1:1000 rabbit, Cell Signalling Technology #4023), p-ERK1/2 (rabbit, Cell Signalling Technology #4370), ERK1/2 (1:500, mouse, Cell Signalling Technology #4696), pS6 (1:1000, rabbit, Cell Signalling Technology #4858), S6 (1:1000, mouse, Cell Signalling Technology #2317), GAPDH (1:1000, mouse, Merck #MAB374), Tau (1:5000, rabbit, Dako Agilent #A0024), and pTau Y18 (1:500, mouse, Genetex #GTX54658). Membranes were then washed 5 times with TBS-Tween and incubated in secondary antibody (1:10,000 anti-mouse IRDye800CW, Li-Cor Biosciences #926-32210 and anti-rabbit IRDye680RD, Li-Cor Biosciences #926-68070) within Odyssey (TBS). Fyn activity was measured as a ratio of phosphorylated ERK1/2 or S6 intensity to total ERK1/2 or S6 intensity, respectively. Tau phosphorylation was measured as a ratio of phosphorylated Tau at Y18 to total Tau. GAPDH and total protein level (Revert) were used to normalise the level of each protein.

### Pharmacological control of Fyn activity and liquid–liquid phase separation

Pharmacological inhibition of Fyn activity was performed by incubating neurons with a potent and specific pharmacological inhibitor of the catalytic activity of SFKs [41–43], 3-(4-Chlorophenyl)-1-(1,1-dimethylethyl)-1H-pyrazolo[3,4-d]pyrimidin-4-amine (PP2), or with the related inactive analogue 1-phenyl-1H-pyrazolo[3,4-d]pyrimidin-4-amine (PP3). Both reagents were incubated at 10 μM for 30 min at 37 °C. LLPS cellular condensates of Tau-P301L protein were dissolved by incubating cells with the aliphatic alcohol 1,6-HD [44]. 1,6-HD (2.5%) was prepared in DMEM, and HEK-293T cells were incubated with 1,6-HD media for 10 min before being imaged.

### Single-particle tracking photoactivated localisation microscopy (sptPALM)

Neurons were transfected at 14–15 days in vitro (DIV14-15) using lipofectamine 2000 (Thermo Fisher Scientific) and incubated for 5–7 days prior to imaging. The following constructs were used for neuronal transfections: pEGFP-N1 (Clontech #6085‐1), mCardinal (Addgene #54590), Fyn-mEos2, Fyn-Y531F-mEos2, Fyn-Y420F-mEos2 (Fyn-mEos2 containing the Y420F mutation), Fyn-K299M-mEos2 (Fyn-mEos2 containing the K299M mutation), Fyn-Y531F-K299M-mEos2, Fyn-ΔSH3-mEos2 (Fyn-mEos2 lacking the SH3 domain, amino-acids 82–143), Tau-P301L-GFP [27] (human tau 2N4R, htau40, containing the FTLD mutation P301L, fused with EGFP at the C-terminus), Δtau74-GFP [22] (human tau 2N4R lacking the MTBR and the C-terminal region, amino-acids 256–441, fused to green fluorescent protein (GFP)). HEK-293T cells were transfected using lipofectamine 3000 (Thermo Fisher Scientific), and incubated for 24–48 h prior to imaging. The following constructs were used for HEK-293T cells transfections: Fyn-mEos2, Fyn-Y531F-mEos2, Fyn-Y531F-K299M-mEos2, MB-anti-SH3-mEos2 (G9 monobody fused with mEos2 at the C-terminus), NB-anti-GFP-mEos2 [45] (nanobody anti-GFP fused with mEos2 at the C-terminus), Fyn-GFP [7] (human full-length Fyn, isoform 1, fused with EGFP at the C-teminus), Tau-P301L-GFP, Tau-P301L-PXXP-GFP (Tau-P301L-GFP containing the P216A and P219A mutations), and Δtau74-GFP.

Imaging acquisitions (16,000 frames at 50 Hz) were taken at 37 °C on an inverted microscope (Nikon) equipped with azimuthal total internal reflection fluorescence (iLas [2], Roper Scientific), a CFI Apo 100×/1.49 NA oil-immersion objective (Nikon Instruments) and an evolve 512 Delta EMCCD camera (Photometrics). A Perfect Focus System (Nikon) was used to avoid axial drifts. MetaMorph software (version 7.10.2, Molecular Devices) was used to steer the sptPALM acquisition. An ultra-flat quadruple beam splitter (ZT405/488/561/647rpc; Chroma Technology) for distortion-free reflection of lasers and a QUAD emission filter (ZET405/488/561/640m; Chroma) were used. Transfected neurons were identified based on their GFP transfection using excitation with a 491 nm laser, or based on their mCardinal transfection using excitation with a 640 nm laser. For sptPALM, Fyn-mEos2 was photoactivated with low-level 405 nm laser excitation (100 mW Vortran Laser Technology). Photoactivated Fyn-mEos2 molecules were subsequently photoconverted using a 561 nm laser (150 mW Cobolt Jive).

### Single-particle trajectory and nanocluster analysis

Tracking of single Fyn-mEos2 molecules was performed in accordance with previous publication [46] using PALM-Tracer software that operates in MetaMorph (Molecular Devices). A wavelet-based segmentation was used to detect and localise single molecules of photoconverted Fyn-mEos2 with nanometric precision [47]. Tracks of Fyn-mEos2 were computed by connecting the localisations using a simulated annealing algorithm. Tracks lasting a minimum of eight frames were reconstructed and used to calculate the mean-square displacement (MSD) of Fyn-mEos2 using the equation MSD (*t*) = *a* + 4*Dt*, where *D* is the diffusion coefficient (in µm^2^/s), *a* = *y* intercept and *t* = time. Trajectories with Log_10_[*D*] > −1.6 were considered mobile. This allowed us to calculate the relative portion of mobile to immobile Fyn-mEos2 molecules from the frequency distribution histogram of Log_10_[*D*] [48, 49].

The clustering of individually tracked Fyn-mEos2 molecules in live cells was quantified and visualised from sptPALM data converted to a simple trajectory number, *x* coordinate, *y* coordinate, acquisition time (TRXYT) format using a custom MATLAB script. TRXYT data were analysed using additional custom Python (version 3.9.6) scripting that utilises the Density-Based Spatial Clustering of Applications with Noise (DBSCAN) functionality of the Python SciKit-Learn module (scikit-learn.org). DBSCAN segments N-dimensional point data into clusters based on a density threshold. For DBSCAN of fixed PALM, the point data traditionally consists of all detected localisations. However, in this study involving live cells, individual sptPALM localisations were not used as point data for DBSCAN because of the bias in the output clustering values towards molecules detected multiple times. Instead, DBSCAN was performed using the spatial centroids of the localisations associated with each molecular trajectory. This ensured that each molecule was only considered once, and that the resulting clustering was more representative of the spatial proximity of molecules in live cells. Spatial centroids for each trajectory (with a minimum of eight localisation steps) in a region of interest were clustered using DBSCAN with empirically determined values of ε = 0.1 µm (radius around each centroid to check for other centroids) and MinPts = 3 (minimum number of centroids within this radius to be considered a cluster). For each DBSCAN cluster, a convex hull of all the localisations comprising the clustered trajectories was used to determine the cluster area.

### FRAP imaging and analysis

HEK-293T cells or hippocampal neurons expressing mCherry and Tau-GFP were imaged using an LSM 710 Inverted point-scanning laser confocal microscope with a 100×/1.4 NA oil objective, equipped with spectral detection and high-sensitivity BiG (GaAsP) detectors, and CO_2_ and temperature controllers. Cells were excited with a 488 nm Argon laser. The FRAP protocol was divided into three sections. Initially, cells were imaged five times every 1.16 s (prebleach). Then, a small selected circular region (2.108 μm ∅) containing Tau-GFP was photobleached, using a 488 nm Argon laser at 80% (dwell time: 35 ms, 100 repeats). After the bleaching, the same region was scanned 50 times every 1.16 s (postbleach). FIJI-ImageJ software was used to measure intensities from the acquisitions. For each time point, the whole-cell fluorescent intensity (*I*_whole_), the background fluorescent intensity (*I*_background_) and the photobleached fluorescence recovery (*I*_FRAP_) integrated density were measured. Recovery intensities were first normalised to the average prebleach fluorescence intensity. Whole-cell and recovery intensities were corrected by subtracting background intensities. Finally, whole-cell intensities were used to correct the recovery intensities, needed to correct for photobleaching due to the acquisition protocol. Equation (1) was used to calculate FRAP curves.

1 $$I_{{{{{{\rm{FRAP}}}}}}\;{{{{{\rm{norm}}}}}}}\left( t \right) = \frac{{I_{{{{{{\rm{whole - prebleach}}}}}}}}}{{I_{{{{{{\rm{whole}}}}}}}\left( t \right) - I_{{{{{{\rm{background}}}}}}}\left( t \right)}} \times \frac{{I_{{{{{{\rm{FRAP}}}}}}}\left( t \right) - I_{{{{{{\rm{background}}}}}}}\left( t \right)}}{{I_{{{{{{\rm{FRAP - prebleach}}}}}}}}}$$

Once normalised intensities were calculated at each time point, averages and SEM values were plotted using GraphPadPrism (version 9.4.1) and the resulting FRAP curve was fitted using nonlinear regression to a one-phase decay curve, obtaining the fluorescence intensity at the plateau (*I*_*∞*_) and the fluorescence intensity at the postbleach point (*I*_0_). The fluorescence intensity at the prebleach point (*I*_*i*_) was used to calculate the mobile fraction (*Mf*) following equation (2).

2 $$Mf = \frac{{I}_{\infty}-{I}_{0}}{{I}_{i} - {I}_{0}}$$

### Statistical analysis

**Fig. 2:**
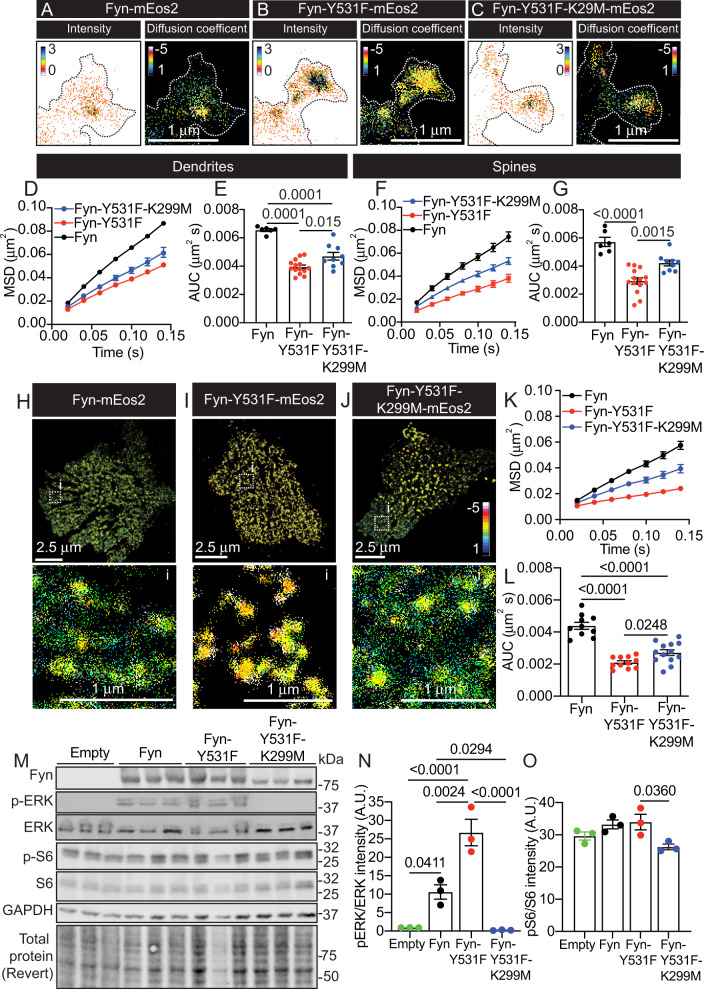
Dephosphorylation of the Y531 epitope controls the lateral entrapment of Fyn-mEos2, leading to downstream activation of ERK/S6 signalling. **A**–**C** Representative intensity and diffusion coefficient maps for **A** Fyn-mEos2, **B** Fyn-Y531F-mEos2 and **C** Fyn-Y531F-K299M-mEos2 within spines of hippocampal neurons. Note that cooler colours within the intensity and the diffusion coefficient maps designate regions of higher localisation intensities and mobility, respectively. The surface of the spines is outlined for visibility. **D**–**G** Mobility of Fyn-mEos2, Fyn-Y531F-mEos2 and Fyn-Y531F-K299M-mEos2 in **D, E** dendrites and **F**, **G** spines, shown as **D**, **F** the MSD (µm^2^) and **E**, **G** area under the curve (AUC) of the corresponding MSD (µm^2^ s). **H**–**J** Diffusion coefficient maps of **H** Fyn-mEos2, **I** Fyn-Y531F-mEos2 and **J** Fyn-Y531F-K299M-mEos2 expressed in HEK-293T cells. Boxed outlines are shown magnified below in (i). Note that hotter colours within diffusion coefficient maps designate regions of lower mobility. Corresponding **K** MSD curve (µm^2^) and **L** AUC of the MSD (µm^2^ s). **M** Western blot of HEK-293T cells transfected with either mEos2 (empty control), Fyn-mEos2, Fyn-Y531F-mEos2 or Fyn-Y531F-K299M-mEos2. **N** Analysis of ERK1/2 activity and **O** S6 activity measured using the relative intensity of the corresponding western blot bands. Error bars are standard errors of the mean (SEM). Mean ± SEM values in **D**–**G** were obtained from neurons co-transfected with mCardinal and Fyn-mEos2 (*N* = 8), Fyn-Y531F-mEos2 (*N* = 14) or Y531F-K299M-mEos2 (*N* = 9). Mean ± SEM values in **K**, **L** were obtained from HEK-293T cells transfected with Fyn-mEos2 (*N* = 10), Fyn-Y531F-mEos2 (*N* = 11) or Y531F-K299M-mEos2 (*N* = 13). Mean ± SEM values in **N**, **O** were obtained from *N* = 3. Statistical comparisons were performed using a one-way ANOVA and Dunnett T3 test for multiple comparisons between groups in **E**, **G** and **L**; or were performed using a one-way ANOVA and Tukey’s test for multiple comparisons between groups in **N** and **O**. The specific adjusted *p* values accounting for multiple comparisons are reported for data considered significantly different (*p* < 0.05).

**Fig. 3:**
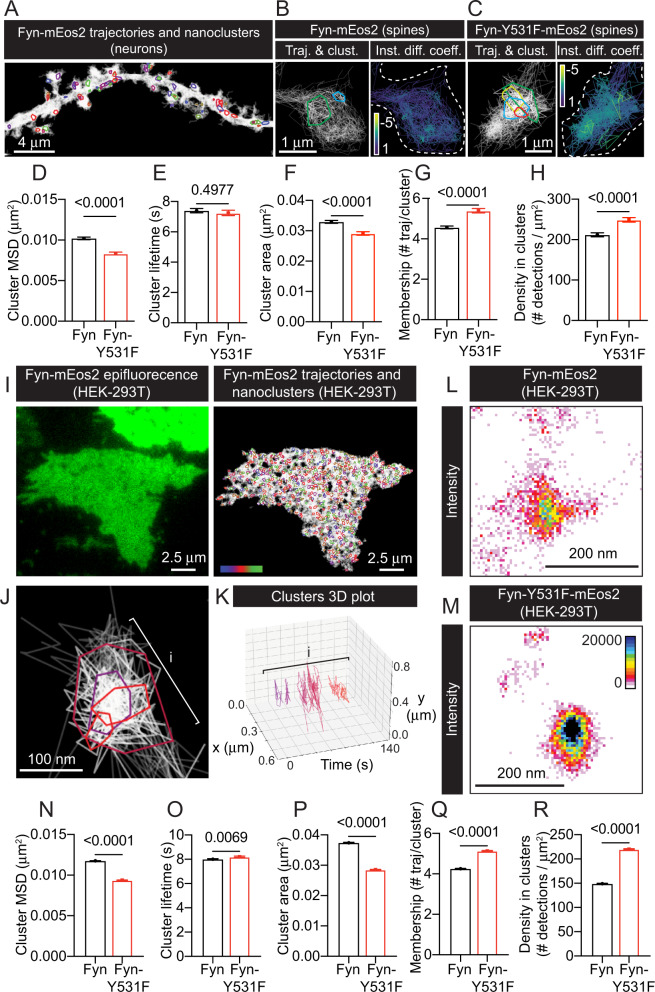
Fyn is organised into nanoclusters in dendritic spines of hippocampal neurons and in HEK-293T cells. **A** Representative image obtained after analysing the spatiotemporal distribution of Fyn-mEos2 trajectories and nanoclusters in hippocampal neurons using NASTIC. **B**, **C** Representative images plotting individual trajectories and clusters or individual trajectories coloured based on their instant diffusion coefficients ([D], with more immobile trajectories depicted in light colours, and more mobile trajectories depicted in dark colours), from **B** Fyn-mEos2 or **C** Fyn-Y531F-mEos2 in dendritic spines. **D** MSD of clustered trajectories (µm^2^). **E** Cluster lifetime (s). **F** Cluster area (µm^2^). **G** Cluster membership (# trajectories/cluster). **H** Density within clusters (# detections/µm^2^). **I** Representative image of Fyn-mEos2 epifluorescence in HEK-293T cells and the corresponding presentation of the spatiotemporal distribution of Fyn-mEos2 trajectories and their nanoclusters. Colour-coding of the clusters is based on their appearance in time across the acquisition (16,000 frames, 320 s). **J** Detail of an area (i) containing Fyn-mEos2 trajectories organised in multiple nanoclusters. **K** 3D (X, Y, Time) plot of Fyn-mEos2 trajectories from the region (i) in (**J**). Squares in X and Y represent 100 nm; squares in Time represent 20 s. **L** Representative intensity map of Fyn-mEos2 in a nanocluster of a HEK-293T cell. **M** Representative intensity map of Fyn-Y531F-mEos2 in a nanocluster of a HEK-293T cell. **N** MSD of clustered trajectories (µm^2^). **O** Cluster lifetime (s). **P** Cluster area (µm^2^). **Q** Cluster membership (# trajectories/cluster). **R** Density within clusters (# detections/µm^2^). Error bars are standard errors of the mean (SEM). Mean ± SEM values in **D**–**H** were obtained from NASTIC analysis of Fyn-mEos2 trajectories (*N* = 1495), and Fyn-Y531F-mEos2 trajectories (*N* = 962) from hippocampal neurons. Mean ± SEM values in **N**–**R** were obtained from NASTIC analysis of Fyn-mEos2 trajectories (*N* = 18,103), Fyn-Y531F-mEos2 (*N* = 20,434) from HEK-293T cells. Statistical comparisons in **D**–**H** and **N**–**R** were performed using unpaired Welch’s *t*-test. The specific adjusted *p* values accounting for the comparisons are reported when the data are considered significantly different (*p* < 0.05).

**Fig. 4:**
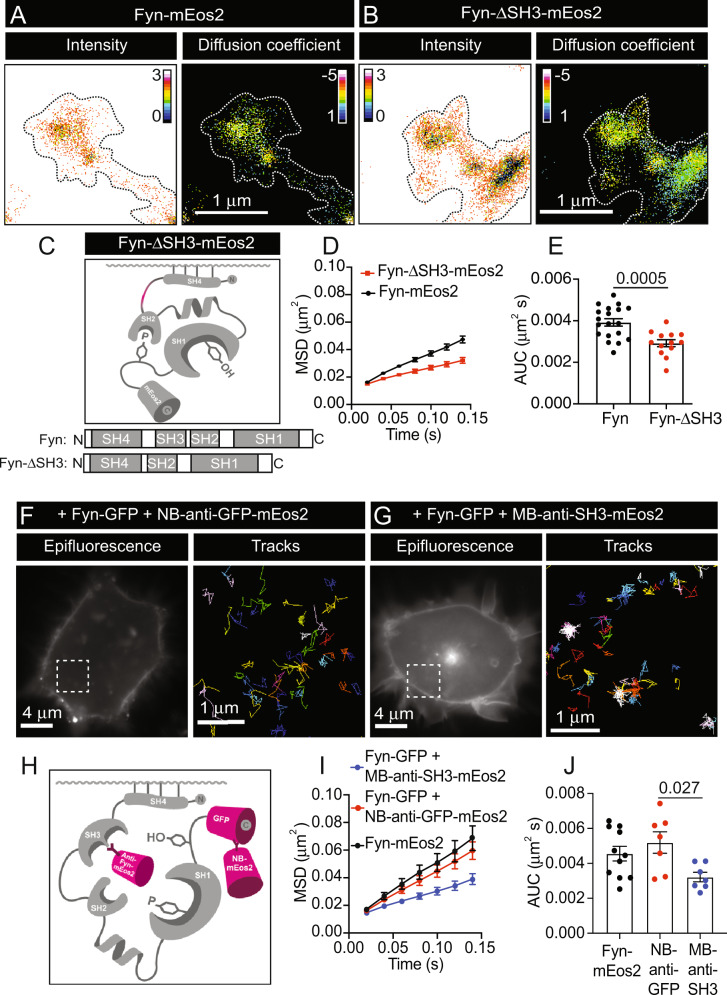
Alteration of the SH3 domain promotes the lateral trapping of Fyn-mEos2. **A**, **B** Representative intensity and diffusion coefficient maps of **A** Fyn-mEos2 or **B** Fyn-ΔSH3-mEos2 in dendrites of hippocampal neurons (DIV18-22). **C** Schematic representation of the tertiary structure of mEos2-tagged Fyn lacking its SH3 domain (Fyn-ΔSH3-mEos2), with a comparison of the domains of full-length Fyn and Fyn-ΔSH3 shown below. **D** Mobility of Fyn-mEos2 and Fyn-ΔSH3-mEos2 indicated as the MSD (µm^2^) curves over time (0.14 s). **E** Corresponding AUC (µm^2^ s) of the MSD graph in **D**. **F** Representative image of Fyn-GFP epifluorescence in HEK-293T cells co-transfected with an mEos2-tagged anti-GFP nanobody (NB-anti-GFP-mEos2). The white box outline is shown at a higher magnification on the right to show individual NB-anti-GFP-mEos2 trajectories. **G** Representative image of Fyn-GFP epifluorescence in HEK-293T cells co-transfected with an mEos2-tagged anti-SH3 monobody (MB-anti-SH3-mEos2). The white box outline is shown at a higher magnification on the right to show individual MB-anti-SH3-mEos2 trajectories. **H** Schematic representation of the binding of anti-GFP-mEos2 nanobodies to the GFP of Fyn-GFP, and anti-Fyn-mEos2 monobodies to the SH3 domain of Fyn-GFP. **I** Mobility of Fyn-mEos2, NB-anti-GFP-mEos2 bound to Fyn-GFP, and MB-anti-SH3-mEos2 bound to Fyn-GFP indicated as the MSD (µm^2^) curves over time (0.14 s). **J** Corresponding AUC (µm^2^ s) of the graphs in **I**. Error bars are standard errors of the mean (SEM). Mean ± SEM values in **D**, **E** were obtained from hippocampal neurons transfected with mCardinal and Fyn-mEos2 (*N* = 19) or Fyn-ΔSH3-mEos2 (*N* = 13). Mean ± SEM values in **I**, **J** were obtained from HEK-293T cells transfected with Fyn-mEos2 (*N* = 11), Fyn-GFP and NB-anti-GFP-mEos2 (*N* = 7), or Fyn-GFP and MB-anti-SH3-mEos2 (*N* = 7). Statistical comparisons were performed using Welch’s *t*-test in **E**, and one-way ANOVA and Tukey’s test for multiple comparisons between groups in **J**. The specific adjusted *p* values accounting for multiple comparisons are reported when the data are considered significantly different (*p* < 0.05).

**Fig. 5:**
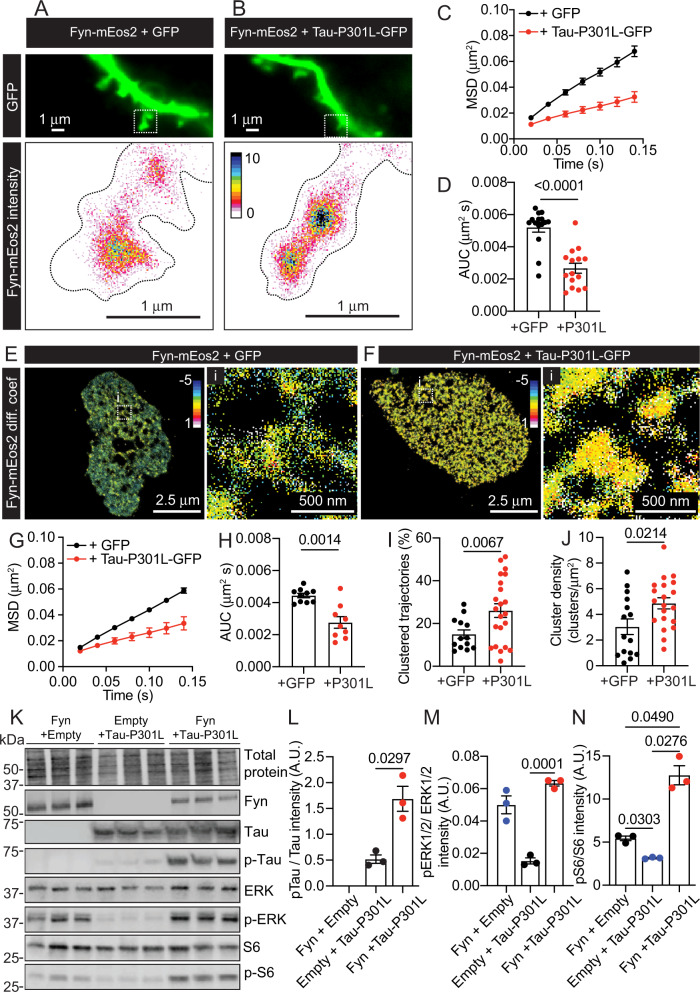
The FTLD P301L mutant Tau promotes the lateral trapping of Fyn-mEos2 in neurons and HEK-293T cells, altering the downstream ERK/S6 signalling. **A** Representative epifluorescence image of GFP co-expressed in hippocampal dendrites with Fyn-mEos2. The white box outline is shown at a higher magnification below to display the intensity map of Fyn-mEos2. **B** Representative epifluorescence image of Tau-P301L-GFP co-expressed in hippocampal dendrites with Fyn-mEos2. The white box outline is shown at a higher magnification below to display the intensity map of Fyn-mEos2. **C** Mobility of Fyn-mEos2 co-expressed with GFP or with Tau-P301L-GFP in hippocampal neurons indicated as the MSD (µm^2^) curves over time (0.14 s). **D** Corresponding AUC (µm^2^ s) of the graphs in **C**. **E**, **F** Representative diffusion coefficient maps of Fyn-mEos2 co-expressed with **E** GFP, or **F** Tau-P301L-GFP in HEK-293T cells. White boxed outlines are shown magnified on the right in (i). Note that hotter colours within diffusion coefficient maps designate regions of lower mobility. **G** Mobility of Fyn-mEos2 co-expressed with GFP or with Tau-P301L-GFP in HEK-293T cells indicated as the MSD (µm^2^) curves over time (0.14 s). **H** Corresponding AUC (µm^2^ s) of the graphs in **G**. **I** Quantification of the % of clustered Fyn-mEos2 trajectories upon co-expression with GFP or with Tau-p301L-GFP. **J** Quantification of the density of Fyn-mEos2 clusters (clusters/μm^2^) upon co-expression with GFP or with Tau-p301L-GFP. **K** Western blot of HEK-293T cells transfected with either Fyn-myc and an empty vector, Tau-P301L-V5 and an empty vector, or Fyn-myc and Tau-P301L-V5. **L** Analysis of Tau phosphorylation at the Y18 epitope (pTau/Tau), **M** ERK1/2 activity (pERK/ERK) and **N** S6 activity (pS6/S6) measured using the relative intensity of the corresponding western blot bands. Error bars are standard errors of the mean (SEM). Mean ± SEM values in **C**, **D** were obtained from hippocampal neurons transfected with Fyn-mEos2 and GFP (*N* = 15) or Tau-P301L-GFP (*N* = 15). Mean ± SEM values in **G**–**J** were obtained from HEK-293T cells transfected with Fyn-mEos2 and GFP (*N* = 10 in **G**, **H** and *N* = 13 in **I**, **J**) or Tau-P301L-GFP (*N* = 9 in **G**, **H** and *N* = 22 in **I**, **J**). Mean ± SEM values in **L**–**N** were obtained from *N* = 3. Statistical comparisons in **D**, **H**, **I** and **J** were performed using unpaired Welch’s *t*-test. Statistical comparisons in **L**–**N** were performed using a one-way ANOVA and Tukey’s test for multiple comparisons between groups. The specific adjusted *p* values accounting for multiple comparisons are reported when the data are considered significantly different (*p* < 0.05).

**Fig. 6:**
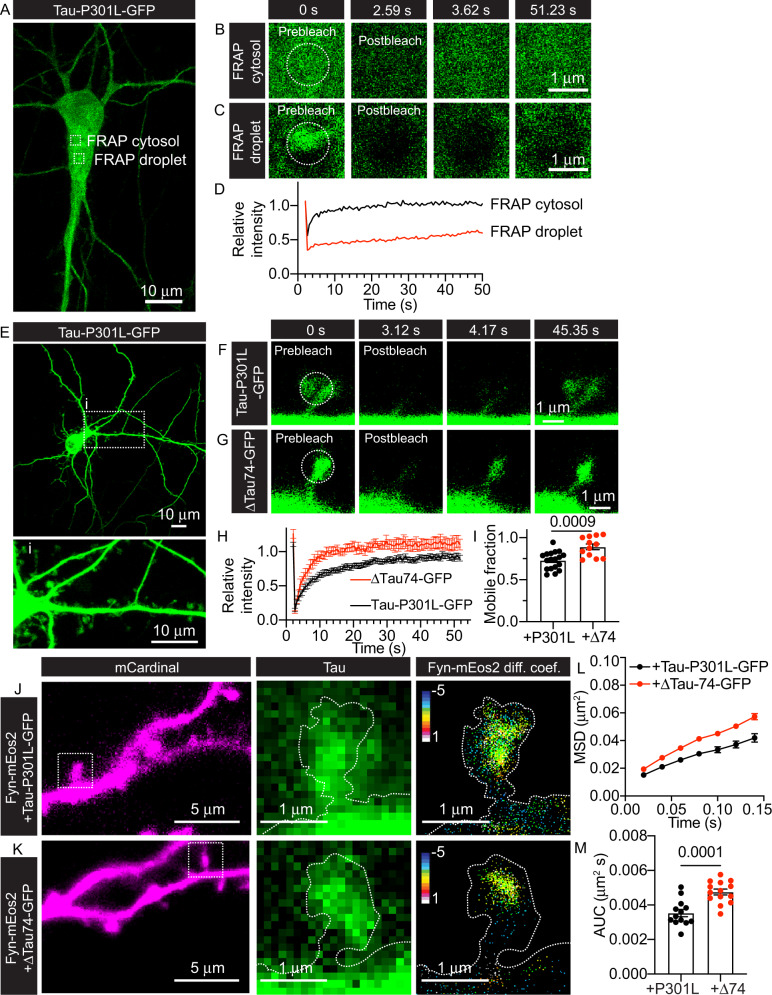
FRAP analysis of FTLD P301L mutant Tau shows slow recovery, but the deletion of the MTBD increases Tau mobility and reverts Fyn-mEos2 lateral trapping in neurons. **A** Representative image of a hippocampal neuron transfected with Tau-P301L-GFP that has an intracellular Tau droplet. White box outlines indicate the cytosolic region and droplet-containing region where FRAP was performed, shown at a higher magnification in **B** and **C**, respectively. **B**, **C** Series of images acquired during FRAP analysis (prebleach 0 s, postbleach 2.59 s, 3.62 s and 51.23 s) of Tau-P301L-GFP in **B** the cytosol or **C** droplet outlined in **A**. **D** Plot of Tau-P301L-GFP FRAP analysis in the cytosol and droplet, as indicated. **E** Representative image of a hippocampal neuron transfected with Tau-P301L-GFP. The white box outline denoted by i is shown magnified below in (i). **F**, **G** Series of images acquired during FRAP analysis (prebleach 0 s, postbleach 3.12 s, 4.17 s and 45.35 s) of **F** Tau-P301L-GFP or **G** ΔTau74-GFP within dendritic spines. **H** Average plot of FRAP analysis of Tau-P301L-GFP or ΔTau74-GFP in dendritic spines, as indicated. **I** Quantification of the mobile fraction of the FRAP curves in **H**. **J** Representative epifluorescence images of mCardinal co-expressed in hippocampal dendrites with Fyn-mEos2 and Tau-P301L-GFP. White box outlines are shown at a higher magnification on the right with Tau-P301L-GFP epifluorescence and corresponding Fyn-mEos2 diffusion coefficient maps shown. Note that hotter colours within the diffusion coefficient map designate regions of lower mobility. **K** Representative epifluorescence images of mCardinal co-expressed in hippocampal dendrites with Fyn-mEos2 and ΔTau74-GFP. White box outlines are shown at a higher magnification on the right with ΔTau74-GFP epifluorescence and corresponding Fyn-mEos2 diffusion coefficient maps shown. Note that hotter colours within the diffusion coefficient map designate regions of lower mobility. **L** Mobility of Fyn-mEos2 co-expressed with Tau-P301L-GFP or with ΔTau74-GFP in hippocampal neurons indicated as the MSD (µm^2^) curves over time (0.14 s). **M** Corresponding AUC (µm^2^ s) of the graphs in **L**. Error bars are standard errors of the mean (SEM). Mean ± SEM values in **H**, **I** were obtained from hippocampal neurons transfected with Tau-P301L-GFP (*N* = 18) or ΔTau74-GFP (*N* = 12). Mean ± SEM values in **L**, **M** were obtained from hippocampal neurons co-transfected with mCardinal, Fyn-mEos2 and Tau-P301L-GFP (*N* = 13) or mCardinal, Fyn-mEos2 and ΔTau74-GFP (*N* = 14). Statistical comparison in **I** and **M** were performed using the unpaired Welch’s *t*-test. The specific adjusted *p* values accounting for multiple comparisons are reported when the data are considered significantly different (*p* < 0.05).

The D’Agostino and Pearson test was used to test for normality. For statistical analysis between two groups of normally distributed data, Student’s *t*-test or Welch’s *t*-test were used. For multiple comparisons, a one-way ANOVA was used with corrections for multiple comparisons. Statistical comparisons were performed on a per-cell basis (Figs. 2 and 4–6 and Supplementary Figs. S1, S2, S4, S5C, E, G, H, S6 and S7G, H), per-cluster basis (Fig. 3 and Supplementary Fig. S3) or per-dish basis (Supplementary Figs. S5B and S7D). The neurons analysed for each experiment were derived from the brains of more than five embryos. The results were obtained from at least two independent experiments. Values are represented as the mean ± SEM. The relevant statistical tests used are indicated in the respective figure legends. For all statistical comparisons, *p* < 0.05 was considered statistically significant, and the numerical *p* value was indicated. Statistical tests were performed, and figures were made using GraphPadPrism (version 9.4.1)

## Results

### The catalytic activity of Fyn does not alter the nanoscale organisation of Fyn in hippocampal dendrites

We recently discovered that within the dendritic spines of hippocampal neurons, Fyn is organised into nanoclusters that are dynamically regulated by neuronal maturation [27]. Here, we first investigated whether changes in Fyn activity affect its nanoscale organisation in live neurons. WT Fyn-mEos2 was expressed in hippocampal neurons, together with mCardinal or GFP as a cytoplasmic marker (Fig. 1D). We used sptPALM in an oblique illumination configuration to image neurons at DIV19-22, selecting secondary dendrites with mature spines for analysis (Fig. 1D). Fyn-mEos2 molecules were stochastically photoconverted from green- to a red-emitting state using 405 nm illumination. Sequences of sparsely distributed photoconverted molecules were acquired at 50 Hz for a duration of 320 s (16,000 frames), localised, and tracked to reveal the nanoscale distribution and dynamics of Fyn (Fig. 1E–G), allowing us to successfully resolved single trajectories of Fyn-mEos2 molecules in individual dendritic spines. The analysis of the trajectories allowed us to compute the frequency distribution of the diffusion coefficients of all the trajectories from the neurons, which was then averaged and grouped into an immobile fraction and a mobile fraction (Fig. 1H). We also computed the MSD of individual trajectories lasting for at least eight frames, and then we calculated the average MSD of all the trajectories from each neuron (Fig. 1I). The average MSD was used in this study for comparison to other conditions.

Given that Fyn has a critical role in integrating a multitude of signalling pathways in neurons [39], we first sought to determine whether its catalytic activity was involved in promoting its nanoclustering at the postsynapse. Based on experiments performed with other SFKs, it has been established that mutations of specific residues within the SH1 domain have profound effects on the activity of these enzymes [2, 50, 51]. Trans-autophosphorylation of the Y420 residue located at the central ‘activation loop’ of the catalytic domain of Fyn (Fig. 1Aii) is required for the transition of Fyn from an open-primed state to an open-active form that is able to phosphorylate its substrates [4, 51]. To determine if autophosphorylation of the Y420 residue within the catalytic SH1 domain controls Fyn nanoclustering, we introduced a phospho-inhibitory mutation (Y420F) to generate a ‘kinase-inactivated’ enzyme (Fyn-Y420F-mEos2) (Supplementary Fig. S1A, B). Our results revealed that this mutation did not affect the mobility of Fyn in secondary dendritic branches of hippocampal neurons (Supplementary Fig. S1D, E). Similarly, no changes were observed in the mobility of Fyn-mEos2 in dendritic spines (Supplementary Fig. S1F, G). To further investigate the link between Fyn catalytic activity and mobility, we generated a ‘kinase-dead’ Fyn enzyme by introducing the mutation K299M also located at the SH1 domain (Fig. 1Aii), which is designed to block the interaction of Fyn with ATP, thereby creating an inactive enzyme that is unable to phosphorylate other substrates even after it has been primed and activated (Fyn-K299M-mEos2) (Supplementary Fig. S1C) [42, 52, 53]. The K299M mutation also had no effect on the mobility of Fyn compared to WT Fyn in either dendrites (Supplementary Fig. S1D, E) or spines (Supplementary Fig. S1F, G). We next investigated the effect of blocking Fyn activity by incubating neurons with 3-(4-chlorophenyl)-1-(1,1-dimethylethyl)-1H-pyrazolo[3,4-d]pyrimidin-4-amine (PP2) (10 μM), a potent and specific pharmacological inhibitor of the catalytic activity of SFKs [41–43]. The structurally related inactive analogue 1-phenyl-1H-pyrazolo[3,4-d]pyrimidin-4-amine (PP3) (10 μM) was used as a control (Supplementary Fig. S2) [54]. PP2 is an ATP-competitive inhibitor that interacts with the hydrophobic pocket near the ATP-binding cleft of the SH1 domain (Supplementary Fig. S2A) [55]. No alterations in Fyn-mEos2 mobility were observed in the dendrites of hippocampal neurons in response to either PP2 or PP3 treatment (Supplementary Fig. S2B, C). Taken together, these results support the notion that the catalytic activity of Fyn does not impact its nanoscale organisation at the postsynapse.

### Induction to an open-primed conformation promotes the nanoscale entrapment of Fyn and increases ERK1/2 activity

The closed conformation of Fyn is primarily maintained through phosphorylation of the Y531 epitope in the C-terminal tail (Fig. 1Aii), which interacts with the SH2 domain (Fig. 1B). To constitutively force Fyn into an open conformation, we introduced a phospho-inhibitory mutation at this residue (Y531F). This open-primed conformation rapidly facilitates the trans-autophosphorylation of the Y420 residue that results in a fully active enzyme. In the presence of the Y531F mutation, Fyn is unable to return to its inactive-closed conformation, resulting in a ‘constitutively active’ kinase [7, 56]. To examine the effect of the Y531F-induced constitutively open conformation on the mobility of Fyn, we performed sptPALM of WT Fyn-mEos2 and Fyn-Y531F-mEos2 in hippocampal neurons (Fig. 2A, B). The Y531F mutation significantly reduced Fyn mobility, indicating that the open conformation of Fyn facilitates the nanoscale lateral trapping of Fyn in dendrites (Fig. 2D, E) and spines (Fig. 2F, G). To evaluate whether the kinase activity of Fyn plays a role in its immobilisation, or whether this effect was solely associated with a change in the conformation towards an extended form, we created the double mutant Fyn-Y531F-K299M-mEos2, which remains constitutively opened but with an inactive catalytic domain (Fig. 2C). This double mutant also showed significantly lower mobility than WT Fyn in dendrites (Fig. 2D, E) and spines (Fig. 2F, G). Interestingly, our results revealed an attenuation in the immobilisation of the opened Fyn in the presence of the K299M mutation (Fig. 2D–G). Together, our findings demonstrate that the nanoscale entrapment of Fyn is principally controlled by its entry into an open-primed conformation through dephosphorylation of the Y531 epitope. The K299 epitope also controls Fyn entrapment, but only in the open conformational state.

We further analysed the nanoscale organisation of Fyn-mEos2 at the surface of human embryonic kidney 293T (HEK-293T) cells. This additional expression system has been widely used to study Tau pathology [39, 57–59], and allows for the study of the mobility of molecules without the confinement of the small surface and topology of dendritic spines. SptPALM of Fyn-mEos2, Fyn-Y531F-mEos2 and Fyn-Y531F-K299M-mEos2 in HEK-293T cells (Fig. 2H–J), and their corresponding analysis revealed an immobilisation associated with the constitutively open conformation mutants (Fig. 2K, L). These differences in mobility reflected those found above in neurons (Fig. 2A–G), further validating the notion that switching to an open conformation is conducive to Fyn nanoclustering. In addition to phosphorylating Tau and the NMDA receptor, Fyn has also been shown to have the mitogen-activated protein kinase ERK1/2 as a substrate. ERK1/2 is a serine-threonine kinase responsible for controlling multiple intracellular neuronal signalling cascades [60]. ERK1/2 activation by Fyn in the presence of the Aβ peptide has been shown to cause downstream phosphorylation of the ribosomal protein S6, which in turn promotes the translation of Tau in AD [39]. To determine whether the immobilisation of Fyn was associated with the downstream activation of ERK1/2 and S6, we extracted the total protein lysates from HEK-293T cells expressing WT Fyn or the constitutively open mutants (Fig. 2M). Expression of Fyn-mEos2 and Fyn-Y531F-mEos2 caused an increase in the relative activity of ERK1/2 (phospho-ERK/total ERK) (Fig. 2N). However, it did not elicit a significant corresponding increase in the activity of S6 (phospho-S6/total S6) (Fig. 2O). In accordance with previous findings [39], the kinase-dead (K299M) mutant, which partially rescues the Y531F-induced lateral entrapment of Fyn, completely silenced ERK1/2 activation and significantly reduced the activity of S6 (Fig. 2O).

### Transition to an active, open conformation promotes the nanoclustering of Fyn

Fyn-mEos2 nanoclustering was subsequently analysed to determine if the decreased mobility of the constitutively active, open conformation Fyn (Y531F) was due to an alteration in its nanoscale organisation. To this end, NAnoscale SpatioTemporal Indexing Clustering (NASTIC) [61] was used to identify and quantify Fyn-mEos2 cluster mobility, apparent lifetime, area, membership and density across dendrites and spines of hippocampal neurons (Fig. 3A–H). Fyn-mEos2 nanoclusters were prominently observed in the presence of the Y531F mutation (Fig. 3B, C). Within nanoclusters, the mobility (cluster MSD) of Fyn-Y531F-mEos2 was significantly lower than that of Fyn-mEos2, indicating that an increase in Fyn immobilisation within nanoclusters had occurred as a result of the mutation (Fig. 3D). Although the apparent duration of nanoclusters (cluster lifetime) was not affected by Fyn being in a constitutively open conformation (Fig. 3E), Y531F mutant nanoclusters did have a significantly smaller area (Fig. 3F). In addition, both the total number of Fyn trajectories trapped within the clusters (cluster membership) (Fig. 3G), and the density of Fyn molecules detected within these nanostructures (density in clusters) (Fig. 3H), were increased in the open Y531F Fyn mutant. These results were further reinforced by spatiotemporal cluster analysis of Fyn-mEos2 nanoclusters in HEK-293T cells (Fig. 3I). Individual Fyn clusters were detected iteratively with an apparent lifetime of 8 s (Fig. 3J, K). Similar to what has been observed in neurons, Fyn-mEos2 and Fyn-Y531F-mEos2 nanoclusters were detected in HEK-293T cells (Fig. 3L, M), with Fyn-Y531F-mEos2 molecules being more immobile than those of Fyn-mEos2 molecules (Fig. 3N). In addition, we detected a small increase in the apparent lifetime of Fyn-Y531F clusters in HEK-293T (Fig. 3O), a reduced Fyn-Y531F nanocluster area (Fig. 3P), and an increased membership and density of Fyn-Y531F molecules in nanoclusters (Fig. 3Q, R). In summary, the decreased mobility imparted by forcing Fyn into a constitutively active, open conformation stems from an increase in the proportion of Fyn molecules that form nanoclusters and the density of these nanoclusters. Furthermore, lateral entrapment of Fyn into nanoclusters is associated with downstream signalling and activation of Fyn substrates.

### Alteration of the SH3 domain renders Fyn more immobile in dendrites

The intramolecular interaction that occurs between the SH3 domain and the PPII helix linker plays an essential role in stabilising the inactive, closed conformation of Fyn and of other SFKs [4, 62]. The SH3 domain is also involved in the interaction of Fyn with its substrates such as heterogeneous nuclear ribonucleoprotein A2 (hnRNPA2) [63], posphoinositide-3-kinase regulatory subunit 1 (PIK3R1, p85α) [64], Asp-His-His-Cys (DHHC) motif-containing palmitoyl acyltransferase 5 (DHHC5) [65], WT Tau [66, 67] and P301L FTLD mutant Tau [18]. As activation of Fyn molecules and other SFKs has previously been demonstrated by forcibly shifting the SH3 domain from its native configuration [62, 68], we aimed to explore whether direct alterations of this domain would also affect the nanoscale organisation of Fyn. To investigate this, we performed sptPALM on a Fyn mutant lacking the SH3 domain (Fyn-ΔSH3-mEos2) (Fig. 4A–C). Deletion of the SH3 domain resulted in decreased Fyn mobility (Fig. 4D, E). Our data suggest that, similarly to the Y531F mutation, disruption of the SH3-PPII helix linker interaction via removal of the SH3 domain destabilises the closed conformation of Fyn. Consequently, this facilitates the transition of Fyn to an open-primed conformation, with increased immobilisation.

Due to the importance of the SH3-PPII helix linker interaction in stabilising the closed conformation of SFKs, several strategies that use small molecule compounds to selectively disrupt the SH3-PPII helix interaction have been developed to manipulate the activity of these enzymes [5, 62, 69, 70]. An example of this is the G9 monobody which interacts with the SH3 domain, displaying high specificity for Fyn compared to other SFKs [71]. To further evaluate how alterations in the SH3-PPII helix linker interaction affect the nanoscale organisation of Fyn, we generated an mEos2-tagged version of this G9 monobody (MB-anti-SH3-mEos2) for use with Fyn-GFP. As a control, we used an anti-GFP nanobody tagged with mEos2 (NB-anti-GFP-mEos2) (Fig. 4F–H). The use of anti-GFP nanobodies tagged with mEos2 was previously exploited to perform single-molecule tracking of GFP-tagged proteins in live cells [45]. Fyn-mEos2 alone was used as an additional control to compare the effect of indirectly detecting and quantifying the mobility of Fyn through the use of the monobodies and nanobodies. Co-expression of Fyn-GFP with either the monobody or the nanobody in HEK-293T cells was performed prior to sptPALM (Fig. 4F, G). As anticipated, the mobility of the nanobody control (Fyn-GFP + NB-anti-GFP-mEos2) was similar to that of Fyn-mEos2 alone, validating this approach. In contrast to this, there was a significant decrease in the MSD of the SH3-binding monobody (Fyn-GFP + MB-anti-SH3-mEos2), further demonstrating the importance of the SH3 domain in dictating the nanoscale mobility of Fyn (Fig. 4I, J). Interfering with the SH3-PPII helix linker region may therefore facilitate the acquisition of a conformation that is conducive to the lateral trapping of Fyn molecules.

### FTLD P301L mutant Tau clusters Fyn leading to alterations in ERK/S6 signalling

We have previously shown that the presence of FTLD P301L mutant Tau aberrantly increased Fyn nanoclustering [27]. This pathological Tau mutant binds the SH3 domain of Fyn with higher affinity than WT Tau [18]. To evaluate the effect of P301L mutant Tau on the mobility of Fyn-mEos2, we co-expressed Fyn-mEos2 together with a GFP-tagged human Tau (2N4R, hTau40) protein that contains the FTLD mutation P301L (Tau-P301L-GFP) [27], in hippocampal neurons and performed sptPALM (Fig. 5A, B). In agreement with our previous findings, expression of Tau-P301L-GFP caused a significant decrease in the mobility of Fyn-mEos2 (Fig. 5C, D). Next, by taking advantage of the lack of endogenous Tau expression in HEK-293T cells, we expressed Fyn-mEos2 in HEK-293T cells and analysed its surface mobility (Fig. 5E, F). The presence of Tau-P301L-GFP significantly increased the immobilisation of Fyn-mEos2 (Fig. 5G, H). To identify and evaluate the parameters of Fyn-mEos2 nanoclusters in the presence of Tau-P301L-GFP, we performed spatiotemporal cluster analysis on HEK-239T cells co-expressing Fyn-mEos2 with either GFP or Tau-P301L-GFP. Our results showed that the percentage of clustered Fyn-mEos2 increased in the presence of Tau-P301L-GFP (Fig. 5I), as did, the density of Fyn-mEos2 nanoclusters (Fig. 5J). Additional cluster parameters were also analysed (Supplementary Fig. S3A–G). The mobility of Fyn-mEos2 inside the clusters was significantly decreased in the presence of Tau-P301L-GFP (Supplementary Fig. S3C). These Fyn-mEos2 clusters were transient with an apparent duration of ~8.5 s, which increased when Tau-P301L-GFP was present (Supplementary Fig. S3D). In addition, co-expression of Tau-P301L-GFP reduced the area of Fyn-mEos2 nanoclusters (Supplementary Fig. S3E). This mutant form of Tau also increased the number of Fyn-mEos2 trajectories that were trapped in nanoclusters (Supplementary Fig. S3F), and the number of Fyn-mEos2 molecules that were detected within these nanoclusters (Supplementary Fig. S3G). Fyn interacts with Tau through its SH3 domain [18, 19]. In addition, Fyn can interact with Tau through its SH2 domain when Tau is phosphorylated at Y18 [15], an early epitope used as a marker for the formation of NFTs in AD patients [16, 17]. Although the structure of Fyn in association with Tau is not known, our mobility results indicate that Fyn is immobilised in the presence of Tau-P301L, which phenocopies the effect of the constitutively open Fyn-Y531F mutant (Fig. 2) and Fyn with an altered SH3 domain (Fig. 4). Together, this suggests that the P301L mutant Tau drives the nanoclustering of Fyn by displacing the intramolecular SH3-PPII helix linker interaction, thereby stabilising Fyn’s open immobile conformation, which promotes downstream signalling (Fig. 2).

To determine whether expression of Tau-P301L elicited a concomitant increase in Fyn-mediated signalling cascades (ERK/S6), changes in ERK and S6 activation were examined upon co-expression of Fyn with Tau-P301L in HEK-293T cells. The total protein fraction was isolated and blotted from HEK-293T cells transfected with either Fyn-myc + empty vector, Tau-P301L-V5 + empty vector, or both Fyn-myc + Tau-P301L-V5 (Fig. 5K). Similar levels of Fyn-myc + Tau-P301L-V5 were found for all three conditions, and the co-expression of Fyn with Tau-P301L caused a marked increase in Tau phosphorylation at the Y18 epitope (Fig. 5L). In addition, a significant increase in ERK signalling (pERK/total ERK) was observed upon co-expression of Fyn + Tau-P301L, compared to Tau alone (Fig. 5M). The presence of both Fyn and Tau-P301L further potentiated the increase of S6 signalling (pS6/S6) that was observed with Fyn alone (Fig. 5N). Together, our data suggest that the P301L Tau mutant promotes further immobilisation of open conformation Fyn, leading to increased nanoclustering and altered downstream ERK/S6 signalling.

### Fyn immobilisation induced by FTLD P301L mutant Tau depends on the ability of Tau to form LLPS condensates

Tau interacts with Fyn through its P-X-X-P motifs located within its proline-rich region [18]. To investigate the molecular determinants responsible for the immobilisation of Fyn, we mutated specific proline residues (P216A and P219A) from the fifth and sixth P-X-X-P motifs of Tau-P301L (Tau-P301L-PXXP), that have been described as critical for binding to Fyn [19, 22]. Removing these residues cannot completely prevent the Tau-P301L-GFP-induced Fyn immobilisation (Supplementary Fig. S4A–D) that was previously detected (Fig. 5), suggesting that other factors could be involved in Fyn immobilisation.

FTLD P301L mutant Tau displays an enhanced propensity to undergo LLPS, forming dynamic liquid droplets in vitro [35, 36]. Tau droplet-like condensates have also been described in cells [36]. As Tau biomolecular condensates are sensitive to protein concentration and perturbations of multivalent low-affinity interactions [34, 72], we explored the sensitivity of Tau-P301L nanoclusters to these alterations. Expression of Tau-P301L-GFP resulted in the formation of droplets in HEK-293T cells (Supplementary Fig. S5A). Increasing Tau-P301L-GFP expression levels increased the number of cells containing these droplets (Supplementary Fig. S5B), which were also of a larger size (Supplementary Fig. S5C). FRAP kinetics have been used to characterise the properties of Tau condensates [72]. FRAP analysis of Tau-P301L-GFP within the cellular droplets showed a slower recovery compared to cytosolic and microtubule-associated Tau-P301L, indicating that the translational diffusion of Tau proteins within the condensates was slower (Supplementary Fig. S5D, E). The aliphatic alcohol 1,6-HD has been used to inhibit the weak interactions formed between highly disordered Tau protein domains that underpin the formation of biomolecular condensates [36, 72, 73]. 1,6-HD dissolved Tau-P301L-GFP droplets in HEK-293T cells, as evidenced by their reduced size and fluorescence intensity (Supplementary Fig. S5F–H), thereby confirming that they were biomolecular condensates. To evaluate the effect of Tau-P301L protein concentration on the nanoscale organisation of Fyn, we compared the nanoscale mobility of Fyn-mEos2 for different expression levels of Tau-P301L-GFP (Supplementary Fig. S6A, B). Increasing the level of Tau-P301L-GFP resulted in reduced mobility of Fyn-mEos2 (Supplementary Fig. S6C, D), without affecting the number of Fyn-mEos2 trajectories that were detected (Supplementary Fig. S6E).

The ability of Tau to undergo LLPS depends on its MTBR region [34]. Therefore, we used a truncated form of the human 2N4R Tau isoform lacking the MTBR and the C-terminal region (amino-acids 256–441), fused to GFP (ΔTau74-GFP) [22, 74]. We expressed Tau-P301L-GFP, Tau-P301L-PXXP-GFP and ΔTau74-GFP in HEK-293T cells (Supplementary Fig. S7A–C, respectively). Whereas mutating the two P-X-X-P motifs in Tau-P301L did not affect the formation of Tau biomolecular condensates, removal of the MTBR abolished their formation (Supplementary Fig. S7D). To evaluate how Tau biomolecular condensates influence the nanoscale mobility of Fyn, we performed sptPALM imaging of Fyn-mEos2 in the presence of Tau-P301L-GFP or ΔTau74-GFP (Supplementary Fig. S7E, F). In the absence of Tau molecular condensates, the immobilisation of Fyn induced by Tau-P301L was not observed (Supplementary Fig. S7G, H), demonstrating that Tau-P301L biomolecular condensates are directly responsible for aberrant Fyn nanoclustering.

Under our expression conditions, we could only detect Tau-P301L droplets in a small fraction of hippocampal neurons (2.7%) (Fig. 6A). FRAP analysis of Tau-P301L-GFP droplets in these neurons also revealed a slow recovery, compared with that of cytosolic Tau-P301L-GFP (Fig. 6B–D), suggesting that these Tau-P301L neuronal structures that exhibit a slow diffusion are indeed biomolecular condensates. P301L Tau exhibits a higher phosphorylation state and a reduced interaction with microtubules compared to WT Tau [75]. This microtubule interaction is important for controlling Tau’s neuronal distribution, as evidenced by reduced dendritic localisation of ΔTau74 [7]. Dendritic spines have been reported to be largely devoid of microtubules, which transiently appear upon the induction of synaptic activity [76]. Thus, both Tau-P301L and ΔTau74 would be expected to have similar molecular dynamics within dendritic spines. However, FRAP analyses of these two Tau mutants within dendritic spines (Fig. 6E–G) revealed that only Tau-P301L had significantly lower mobility (Fig. 6H, I). This lower mobility of the FTLD mutant Tau could be associated with transiently formed nascent Tau condensates. SptPALM analysis of the nanoscale organisation of Fyn-mEos2 in spines (Fig. 6J, K) revealed a significant increase in Fyn mobility in the presence of ΔTau74, compared to Tau-P301L (Fig. 6L, M). Overall, our results suggest that the FTLD P301L mutant Tau immobilises Fyn within dendritic spines through the formation of biomolecular condensates.

## Discussion

Here, we unravel the mechanism allowing Fyn to integrate multiple signalling cascades, via the immobilisation of Fyn in nanoclusters. This involves the transition of Fyn from a closed to an open conformation, which facilitates its clustering similar to what has been reported for other SFKs [77]. We demonstrate that this conformational switch not only induces clustering but also leads to increased Fyn/ERK/S6 signalling. This is relevant in the context of Fyn dysregulation which has been associated with neurological disorders such as AD and other Tauopathies including FTLD-Tau [78, 79]. Furthermore, we reveal that pathological FTLD P301L mutant Tau forms biomolecular condensates that locks additional Fyn molecules in an immobile, open conformation, which leads to altered downstream ERK and S6 signalling.

The kinase activity of Fyn and other SFKs is controlled by their transition between two opposing configurations, from a closed/inactive state to an open/primed state. This transition allows SFKs to bind their substrates and execute their catalytic activity [50]. Closed Fyn is stabilised through two intramolecular interactions, one that occurs between the SH2 domain and phosphorylated Y531 residue (in the human sequence) located at the C-terminal tail, and another that occurs between the hydrophobic residues of the SH3 domain and the proline-rich PPII helix linker [2]. Our data show that altering any of these interactions reduces the mobility of Fyn, suggesting that the transition into an open-primed conformation promotes Fyn’s lateral entrapment in nanoclusters. Opening of SFKs has been reported to confer their catalytic activity because in this conformation, the SH2 and SH3 domains become accessible to external ligands [5], and the SH1 catalytic domain can be activated immediately following trans-autophosphorylation of the Y420 residue [4]. Interestingly, we found that activation of the catalytic SH1 domain has little impact on the mobility of Fyn. Indeed, neither pharmacological suppression (PP2 treatment) nor genetic inhibition (Y420F and ‘kinase-dead’ K299M mutants) impacted Fyn mobility. By contrast, the creation of an open-but-inactive Fyn through the addition of the K299M mutation in the background of the ‘open’ Y531F mutant partially rescued Fyn immobilisation. The nanoclustering and activation of Fyn likely undergo a two-step process: firstly, Fyn acquires an open conformation that initiates its organisation into nanoclusters; then the opened Fyn is activated via autophosphorylation of the Y420, further locking Fyn in the nanoclusters. This two-step model explains why the K299M and Y420F mutations within the catalytic SH1 domain alone do not affect cluster formation. Only by opening Fyn into its extended conformation is the SH1 domain finally exposed to its substrates, triggering efficient downstream signalling.

We have previously reported that Fyn is organised in compacted nanodomains in dendrites [27]. Molecular crowding [80, 81], variable spine geometry [82] and interaction of Fyn with neighbouring proteins may regulate this entrapment process. Critically, the opening of Fyn molecules exposes their SH2 and SH3 motifs [3], which promotes lateral trapping of Fyn into nanoclusters. However, it is unclear which interactions are responsible for this effect. Self-association has been described for other SFKs, with the formation of dimers enabling rapid potentiation of their activity when the enzymes adopt an open conformation [83]. This suggests that multimerization of opened, primed Fyn molecules could facilitate their clustering in dendrites. PSD95 is another possible candidate, as it stabilises molecules at the postsynapse and forms nanodomains in spines of size and frequency that is comparable to those observed for Fyn [46, 84]. Both PSD95 and Fyn associate with the plasma membrane upon palmitoylation of distinct residues [1, 6, 85], and PSD95 interacts with the SH2 domain of Fyn [6]. This suggests that entry into an open conformation may induce Fyn’s binding to PSD95, resulting in their postsynaptic nanoclustering.

Nanoclustering has been linked to the increased activity of multiple pre- and postsynaptic molecules, including syntaxin1A [48], Munc18-1 [86] and AMPA receptors [46]. The results of this study highlight the critical synergy that occurs between the nanoclustering and enzymatic activity of Fyn kinase. The constitutively open Fyn mutant (Fyn-Y531F) caused a significant increase in the activity of ERK1/2 and a minor elevation in S6 activity. By contrast, addition of the K299M mutation (K299M-Y531F), which partially alleviated Fyn nanoclustering, completely silenced ERK1/2 and reduced S6 activity. These observations suggest that Fyn nanoclustering and catalytic activity are intrinsically linked, the latter comprising a critical step underpinning the initiation of downstream signalling events. However, nanoclustering in isolation may be insufficient for triggering pathological signalling events, as they may require the engagement with additional interacting proteins. Overexpression of the FTLD P301L mutant Tau led to entrapment of Fyn in nanoclusters and elicited an increase in Fyn’s downstream signalling, specifically promoting pronounced phosphorylation of both the ribosomal S6 kinase [87, 88] and Tau’s pathological residue Y18. Previous findings have identified Fyn as a key modulator of Tau pathologies [9, 89, 90] reinforcing the importance of the Tau/Fyn/ERK/S6 signalling complex in the development and progression of dementias. Our findings shed light on the nanoscale organisation of Fyn and Tau proteins, highlighting the importance of nanocluster formation for the generation of pathological signalling. Further work will be needed to reveal the nanoscale organisation of other downstream proteins and to determine the impact that this has on the regulation of signalling cascades.

The fact that SFKs are broadly expressed and share a common negative regulatory mechanism based on intramolecular interactions involving the SH2-tail and SH3-linker, has motivated extensive research into exploiting these properties. Strategies based on the creation of synthetic SH2/SH3-binding small molecules have been designed to finely control the activity of SFKs [5, 62, 69, 70]. Interestingly, monobodies [69, 71] and peptoid-based ligands [91] that are highly selective for the SH3 domain of Fyn have also been generated to modulate the activity of Fyn kinase. Here we used an mEos2-tagged version of the G9 monobody, that selectively binds the SH3 domain of Fyn [71]. As Fyn interacts with phosphorylated Tau through its SH2 and SH3 domains [18, 19, 66], it is possible that binding of our G9-mEos2 monobody to the SH3 domain could induce a steric effect that interferes with the conformational mobility of Fyn. Our results indicate that binding of the G9-mEos2 monobody reduces the mobility of Fyn molecules, reinforcing the idea that interfering with the intramolecular SH3-linker promotes an open-primed and immobilised Fyn conformation. The equilibrium dissociation constant (K_D_) for G9 monobodies bound to the Fyn SH3 domain, calculated using isothermal titration calorimetry, is 0.166 μM [71]. In a different study using surface plasmon resonance, the K_D_ values calculated for WT Tau and Tau-P301L bound to the SH3 domain of Fyn were 6.77 and 0.16 μM, respectively [18]. These results suggest that G9 monobodies bind to the SH3 domain of Fyn with higher affinity than Tau, and at a level comparable to FTLD P301L mutant Tau. The data are in line with our observation that overexpression of mutant P301L Tau decreases Fyn mobility in a manner similar to G9 monobodies.

The interaction between Tau and Fyn has previously been shown to contribute to neurodegeneration associated with AD [22] and FTLD-Tau [89, 90]. It has been suggested that the manipulation of this interaction could represent a potential therapeutical intervention. Decreasing either Tau [92] or Fyn [12, 93] expression protects against Aβ toxicity in AD. However, undesired effects such as memory deficits associated with reducing Fyn levels [94] highlight the need for further investigation into developing therapeutics with more desirable outcomes. The recent development of a cell-permeable peptide inhibitor of the Fyn-Tau interaction yielded promising results by reducing the endogenous Fyn-Tau interaction and Tau phosphorylation [95]. G9 monobodies have also been used, demonstrating the efficacy of inhibiting Fyn-Tau binding [96]. Our findings on Fyn mobility using G9 monobodies against the SH3 domain are in line with these results.

Tau controls the localisation and nanoclustering of Fyn in dendrites [22, 27]. The interaction between Fyn and Tau has been extensively characterised in terms of the residues responsible for this binding [15, 18, 19, 66] and the impact of their phosphorylation [97]. Interestingly, our findings obtained by mutating the P-X-X-P residues of Tau-P301L involved in Fyn SH3 binding suggest that Fyn nanoclustering is only partially dependent on Fyn-Tau interaction. Instead, the ability of pathogenic Tau-P301L to generate biomolecular condensates through LLPS represents a tantalising new mechanism that could be involved in the immobilisation and clustering of Fyn. Indeed, LLPS have been recently suggested to be involved in clustering of cellular structures [98, 99]. The fact that the Tau-P301L mutant lacking the P-X-X-P residues can still form cellular condensates and immobilise Fyn, suggests that Tau-LLPS-dependent cellular compartmentalisation favours the nanoclustering of Fyn. Although it is conceivable that Fyn nanoclustering could be facilitated by additional interactors, our results from both neurons and HEK-293T cells point to a model where Tau-P301L biomolecular condensates are sufficient to immobilise Fyn. We thus suggest a mechanistic model where the ability of Tau-P301L to undergo LLPS promotes membraneless biomolecular condensates that operate as subcellular ‘hubs’ where crowded Fyn molecules may remain immobile, ordered and compartmentalised, facilitating their interaction with downstream signalling proteins. To date, we lack detailed structural information about how Fyn and Tau interact. However, it is tempting to speculate that both WT and P301L mutant Tau may preferentially interact with and stabilise Fyn in its open conformation. Importantly, further experiments are required to determine whether the entrapment of Fyn in condensates can induce a conformational change that extends the structure of Fyn, transitioning to an open state primed for Tau interaction.

In conclusion, we demonstrate that Fyn nanoclustering is exacerbated by FTLD P301L mutant Tau biomolecular condensates and is controlled by the phosphorylation-dependent switch of Fyn to an open conformation which occurs independently of its catalytic activation. This nanoclustering leads to enhanced intracellular, downstream ERK1/2 and S6 signalling, a pathway implicated in AD and FTD [39]. Overall, our results support the idea that the ability of Tau to associate with Fyn in FTLD-Tau alters the nanoscale organisation of Fyn and consequently its physiological function, with potential long-term ramifications for neurodegeneration. Finally, the demonstration that pathogenic Tau biomolecular condensates confer further nanoclustering of Fyn opens the way to designing novel therapeutical interventions based on selectively inhibiting toxic Tau condensates in order to re-establish proper postsynaptic Fyn clustering and signalling.

## Supplementary information

Supplementary Information

## Data Availability

All data needed to evaluate the conclusions in the paper are present in the paper and/or the Supplementary materials.
