# Supplementary material for: Fyn nanoclustering requires switching to an open conformation and is enhanced by FTLD-Tau biomolecular condensates
Source: Mol Psychiatry. 2022 Oct 18;28(2):946–62. doi: 10.1038/s41380-022-01825-y (PMC9908554; doi:10.1038/s41380-022-01825-y)
Supplement: Supplementary file 1 — Supplementary Information [file 41380_2022_1825_MOESM1_ESM.docx]

**Supplementary Information (SI)**

**Supplementary Figure S1.**

**Supplementary Figure S1. Inhibition of the catalytic (SH1) domain of Fyn does not impact the mobility of Fyn-mEos2.**

(**A**) Intensity and diffusion coefficient map for Fyn-mEos2 in a dendritic spine of a representative hippocampal neuron. (**B**) Intensity and diffusion coefficient map for Fyn-Y420F-mEos2 in a dendritic spine of a representative hippocampal neuron. (**C**) Intensity and diffusion coefficient map for Fyn-K299M-mEos2 in a dendritic spine of a representative hippocampal neuron. Note that cooler colours within the intensity and the diffusion coefficient maps in A-C designate regions of higher localization intensities and mobility, respectively. (**D, F**) Mobility of Fyn-mEos2, Fyn-Y420F-mEos2 and Fyn-K299M-mEos2 indicated as the MSD (µm^2^) curves over time (0.14 s) in dendrites (D) or in spines (F). (**E, G**) Corresponding AUC [(µm^2^ s) x 100] of the graphs in D and F. Error bars are standard errors of the mean (SEM). Mean ± SEM values were obtained from hippocampal neurons transfected with mCardinal and Fyn-mEos2 (N = 14), Fyn-K299M-mEos2 (N=12) or Fyn-Y420F-mEos2 (N=8). Statistical comparisons were performed using a one-way ANOVA and Tukey’s *post-hoc* test for multiple comparisons. Although no statistically significant differences were observed, the specific adjusted *p* values accounting for multiple comparisons are shown.

**Supplementary Figure S2.**

**Supplementary Figure S2. Pharmacological inhibition of the catalytic activity of Fyn with pyrazolopyrimidine 2 (PP2) does not impact the mobility of Fyn-mEos2.**

(**A**) Illustration depicting the pharmacological inhibition of Fyn (grey) with PP2 (purple) while in its closed (left) and open (right) conformation. (**B**) Diffusion coefficient map for Fyn-mEos2 in a dendritic spine of a representative hippocampal neuron treated with either PP3 (control; 10 μM, 30 min) or PP2 (10 μM, 30 min). Note that cooler colours within the diffusion coefficient maps in B designate regions of higher mobility. (**C**) Mobility of Fyn-mEos2 after treatment with either PP3 or PP2, indicated as the AUC (µm^2^ s) of the MSD curves (not shown). Mean ± SEM values were obtained from hippocampal neurons transfected with mCardinal and Fyn-mEos2 and treated with either PP3 (N = 9) or PP2 (N = 11). Statistical comparisons were performed using the Student’s *t*-test. Although no statistically significant differences were observed, the specific adjusted *p* value accounting for the comparison is shown.

**Supplementary Figure S3.**

**Supplementary Figure S3. Expression of the FTLD P301L mutant tau increases the clustering of Fyn-mEos2 in HEK-293T cells.**

(**A**) Representative image showing the spatiotemporal distribution of Fyn-mEos2 trajectories and their nanoclusters in HEK-293T cells co-expressing Fyn-mEos2 and GFP. (**B**) Representative image showing the spatiotemporal distribution of Fyn-mEos2 trajectories and their nanoclusters in HEK-293T cells co-expressing Fyn-mEos2 and Tau-P301L-GFP. Note that individual clusters are coded with a different colour. (**C**) MSD of clustered trajectories (µm^2^ / s). (**D**) Cluster lifetime (s). (**E**) Cluster area (µm^2^). (**F**) Cluster membership (# trajectories / cluster). (**G**) Density within clusters (# detections / µm^2^). Error bars are standard error of the mean (SEM). Mean ± SEM values in C - G were obtained from NASTIC analysis of Fyn-mEos2 trajectories in the presence of either GFP (N = 14281) or Tau-P301L-GFP (N = 32136) in HEK-293T cells. Statistical comparisons in C - G were performed using an unpaired Welch’s *t*-test. The specific adjusted *p* values accounting for the comparisons are shown.

**Supplementary Figure S4.**

**Supplementary Figure S4. Disruption of the tau-Fyn interaction by mutating the P-X-X-P motif of Tau-P301L cannot completely prevent Fyn-mEos2 immobilisation.**

(**A**) Representative images of Tau-P301L-GFP TIRF fluorescence and Fyn-mEos2 diffusion coefficient map, which were co-expressed in HEK-293T cells. The square dotted outline shows the Fyn-mEos2 diffusion coefficient map at a higher magnification (**i**). (**B**) Representative images of Tau-P301L-PXXP-GFP epifluorescence and Fyn-mEos2 diffusion coefficient map, which were co-expressed in HEK-293T cells. The square inset dotted outline shows Fyn-mEos2 diffusion coefficient map at a higher magnification (**i**). Note that cooler colours within the diffusion coefficient maps in A and B designate regions of higher mobility. (**C**) Mobility of Fyn-mEos2 in the presence of either Tau-P301L-GFP or Tau-P301L-PXXP-GFP is indicated as the MSD (µm^2^) curves over time (0.14 s). (**D**) Corresponding AUC (µm^2^ s) of the graph in C. Mean ± SEM values in C, D were obtained from the analysis of Fyn-mEos2 mobility in the presence of either Tau-P301L-GFP (N = 20) or Tau-P301L-PXXP-GFP (N = 16) in HEK-293T cells. Statistical comparison in D was performed using an unpaired Welch’s *t*-test. The specific adjusted *p* value accounting for the comparison is reported.

**Supplementary Figure S5.**

**Supplementary Figure S5. Tau-P301L forms biomolecular condensates in HEK-293T cells.**

(**A**) Representative images of HEK-293T cells transfected with increasing concentrations of Tau-P301L-GFP plasmid, as indicated. Arrowheads indicate the position of Tau-P301L-GFP droplets. (**B**) Percentage of cells containing Tau-P301L-GFP droplets. (**C**) Average size of Tau-P301L-GFP droplets. (**D**) Representative images of HEK-293T cells expressing Tau-P301L-GFP, where FRAP analysis was performed either inside (**i**) or outside (**ii**) Tau-P301L-GFP droplets. White box dotted outlines are shown magnified on the right. The series of images acquired during FRAP analysis show prebleaching, postbleaching and 53.4 s time, with the region of interest shown by a white circle dotted outline. (**E**) FRAP analysis of Tau-P301L-GFP within the cytosol and droplets, as indicated. (**F**) Representative images of HEK-293T cells expressing Tau-P301L-GFP before and after treatment with 1,6-HD (2.5 %, 10 min). Arrowhead indicate the position of a Tau-P301L-GFP condensate. (**G**) Size (µm^2^ ) of Tau-P301L-GFP condensates before and after treatment with 1,6-HD. (**H**) Fluorescent intensity in Tau-P301L-GFP condensates before and after treatment with 1,6-HD. Data in B, C and E are displayed as mean ± SEM. Statistical comparisons in B and C were performed using the Brown-Forsythe and Welch ANOVA test followed by a Dunnett T3 *post-hoc* test comparing the 0.5 µg and 2 µg conditions to the lowest concentration of Tau-P301L-GFP plasmid (0.1 µg). Statistical comparisons were performed on a per-dish basis in B (30 to 200 cells analyzed per dish), or a per-cell basis in C. Statistical comparisons in G and H were performed on a per-cell basis, and a paired Student’s *t*-test was used. The specific adjusted *p* values accounting for the comparisons are reported.

**Supplementary Figure S6.**

**Supplementary Figure S6. Increasing concentrations of Tau-P301L-GFP immobilizes Fyn-mEos2 in HEK-293T cells.**

(**A, B**) Representative images of Tau-P301L-GFP epifluorescence and Fyn-mEos2 diffusion coefficient map, which were co-expressed in HEK-293T cells. 0.5 μg of Tau-P301L-GFP was co-expressed with Fyn-mEos2 in A, and 2 μg of Tau-P301L-GFP was co-expressed with Fyn-mEos2 in B. Note that cooler colours within the diffusion coefficient maps in A and B designate regions of higher mobility. (**C**) Mobility of Fyn-mEos2 in the presence of either 0.5 μg or 2 μg of Tau-P301L-GFP is indicated as the MSD (µm^2^) curves over time (0.14 s). (**D**) Corresponding AUC (µm^2^ s) of the graph in C. (**E**) Quantification of the number of Fyn-mEos2 trajectories per cell. Data in C - E are displayed as mean ± SEM. Statistical comparisons in D and E were performed using the unpaired Welch’s *t*-test. The specific adjusted *p* value accounting for the comparison is reported.

**Supplementary Figure S7.**

**Supplementary Figure S7. Truncation of the MTBR region of tau results in a lack of biomolecular condensate formation, and prevents Fyn-mEos2 immobilisation.**

(**A - C**) Representative images of HEK-293T cells transfected with Tau-P301L-GFP (A), Tau-P301L-PXXP-GFP (B) or ΔTau74-GFP (C). Arrowhead indicates the position of tau droplets. (**D**) Percentage of cells containing tau droplets. (**E**) Representative images of Tau-P301L-GFP epifluorescence and Fyn-mEos2 diffusion coefficient map, which were co-expressed in HEK-293T cells. The square inset (**i**) shows Fyn-mEos2 diffusion coefficient map at a higher magnification (**i**). (**F**) Representative images of Tau- ΔTau74-GFP epifluorescence and Fyn-mEos2 diffusion coefficient map, co-expressed in HEK-293T cells. The square dotted outline shows the Fyn-mEos2 diffusion coefficient map at a higher magnification (**i**). Note that hotter colours within diffusion coefficient maps in E and F designate regions of lower mobility. (**G**) Mobility of Fyn-mEos2 in the presence of either Tau-P301L-GFP or ΔTau74-GFP is indicated as the MSD (µm^2^) curves over time (0.14 s). (**H**) Corresponding AUC (µm^2^ s) of the graph in G. Data in D, G and H are displayed as mean ± SEM. Statistical comparisons in D were performed using the Brown-Forsythe and Welch ANOVA test followed by a Dunnett T3 *post-hoc* test. Statistical comparisons in D were performed on a per-dish basis (36 to 182 cells analyzed per dish). In G and H, Fyn-mEos2 mobility in the presence of either Tau-P301L-GFP (N = 11) or Tau-P301L-PXXP-GFP (N = 11) in HEK-293T cells was analysed. Statistical comparison in H was performed using the unpaired Welch’s *t*-test. The specific adjusted *p* values accounting for the comparison are reported.
